# Generative AI for Bayesian Computation

**DOI:** 10.3390/e27070683

**Published:** 2025-06-26

**Authors:** Nick Polson, Vadim Sokolov

**Affiliations:** 1 Booth School of Business, University of Chicago, Chicago, IL 60637, USA; ngp@chicagobooth.edu; 2 Department of Systems Engineering and Operations Research, George Mason University, Fairfax, VA 22030, USA

**Keywords:** generative AI, Bayesian computation, quantile neural networks, traffic flow, satellite drag

## Abstract

Generative Bayesian Computation (GBC) provides a simulation-based approach to Bayesian inference. A Quantile Neural Network (QNN) is trained to map samples from a base distribution to the posterior distribution. Our method applies equally to parametric and likelihood-free models. By generating a large training dataset of parameter–output pairs inference is recast as a supervised learning problem of non-parametric regression. Generative quantile methods have a number of advantages over traditional approaches such as approximate Bayesian computation (ABC) or GANs. Primarily, quantile architectures are density-free and exploit feature selection using dimensionality reducing summary statistics. To illustrate our methodology, we analyze the classic normal–normal learning model and apply it to two real data problems, modeling traffic speed and building a surrogate model for a satellite drag dataset. We compare our methodology to state-of-the-art approaches. Finally, we conclude with directions for future research.

## 1. Introduction

Generative Bayesian Computation (GBC) provides a simulation-based approach for generating samples from a posterior distribution. To do this, we train a deep neural network to map samples from a base distribution to the posterior distribution, thus avoiding the use of densities. The inverse posterior mapping is learned directly via optimization of parameters of a deep generative quantile map. GBC applies to all forms of inference including both likelihood-free and parametric models for predictions and maximum expected utility analysis [1]. To illustrate our methodology, we provide three examples. First, learning in the normal–normal model and, secondly, we build a surrogate model for a satellite drag dataset (see [2]).

The key idea behind GBC is to start with a large training sample from the joint distribution of observable and parameters, denoted by ${\left(y^{\left(i\right)},\theta^{\left(i\right)}\right)}_{i=1}^{N}$ where training sample size *N* is large, typically N≫n, $y=(y_{1},\ldots ,y_{n})$, $\theta =(\theta_{1},\ldots ,\theta_{d})$. For the data-generating process, we allow for both parameter likelihood inference $y^{\left(i\right)}|\theta^{\left(i\right)}∼p\left(y|\theta^{\left(i\right)}\right)$ and likelihood-free models where $y^{\left(i\right)}=f\left(\theta^{\left(i\right)}\right)$ is a given forward map.

Samples are generated from the prior $\theta^{\left(i\right)}∼\pi \left(\theta \right)$, and a base distribution $\tau^{\left(i\right)}∼p\left(\tau \right)$, typically a vector of uniforms. We can directly find the mapping

$$\theta^{\left(i\right)}=G\left(S\left(y^{\left(i\right)}\right),\tau^{\left(i\right)}\right),$$

Here, *S* is a summary statistic, which is a lower-dimensional representation of *y*. The summary statistic is a sufficient statistic in the Bayes sense [3], and *G* is a generator map to transport the base variable τ to the posterior density θ∣*y*. Both *G* and *S* are deep neural networks estimated from the training dataset. In the univariate case, when the base distribution is uniform, the inverse posterior map is simply given by the inverse posterior cumulative distribution function, namely

$$\theta \overset{D}{=}F_{\theta |y}^{-1}\left(S\left(y\right),\tau \right).$$

Here, *S* is a dimension reducing summary statistic, [4], $y\in R^{n}$, and $\theta \in R^{k}$. In the multi-parameter case, when k>1, we use an RNN or autoregressive structure where we model a vector via a sequence

$$(F_{\theta_{1}|y}^{-1}\left(\tau_{1}\right),F_{\theta_{2}|\theta_{1},y}^{-1}\left(\tau_{2}\right)\;\ldots ),$$

and estimate the quantile functions recursively.

Our work then builds on [4] who were the first to propose deep learners for dimension reduction methods for S(y) and to provide asymptotic theoretical results. We also build on the insight by [5,6] that implicit quantile neural networks can be used to approximated distributions that arise in reinforcement learning. Ref. [7] also provides the connection between the widely used 1-Wasserstein distance and quantile regression.

GBC allows the researcher to learn a dimensionality-reduced summary (a.k.a. sufficient) statistics along with a non-linear map [4,8]. A useful interpretation of the sufficient statistic is as a posterior mean, which also allows us to view posterior inputs as one of the inputs to the posterior mean. One can also view a NN is an approximate non-linear Bayes filter to perform such tasks [9].

Our framework provides a natural link for black box methods and stochastic methods, as commonly known in the machine learning literature [10,11]. Quantile deep neural networks and their ReLU/tanh counterparts provide a natural architecture. Approximation properties of those networks are discussed in [12]. Dimensionality reduction can be performed using autoencoders and partial least-squares [13] due to the result by [10,14] (see survey by [15]), and the kernel embedding approach discussed by [16]. Generative models have been considered in Bayesian analysis by [17]. Quantile generative methods proposed in this paper circumvent the need for methods such as MCMC that require density evaluations. Hence, GBC methods provide the practitioner with a useful simulation-based tool for inference and uncertainty quantification in a variety of applied engineering and scientific scenarios.

### Connections to Previous Work

Generative models often arise in the context of inverse problems [18,19] and decision-making problems [7]. In the context of inverse problems, the prediction of a mean is rarely an option, since the average of several correct values is not necessarily a correct value and might not even be feasible from the physical point of view. The two main approaches are surrogate-based modeling and approximate Bayes computations (ABC) [15,16,20]. Surrogate-based modeling [21] is a general approach to solving inverse problems, which is based on the availability of a forward model y=f(θ), which is a deterministic function of parameters θ. The forward model is used to generate a large sample of pairs (y,θ), which is then used to train a surrogate, typically a Gaussian process, which can be used to calculate the inverse map. For a recent review of the surrogate-based approach, see [18]. There are multiple papers that address different aspects of surrogate-based modeling.

Approximate Bayesian Computation (ABC) methods are a generative approach based on local kernel smoothing. There are two major differences to our approach. One, is how the training dataset is generated. We avoid the use of rejection sampling. The second difference is the use of a quantile neural network (QNN) to approximate the posterior distribution.

In our framework, the map *G* can be viewed as a nearest neighbor network. ABC relies on the comparison of the summary statistic *S* calculated from the observed data with the summary statistics calculated from the simulated data. Ref. [22] show that a natural choice of *S* is via the posterior mean. Ref. [23] shows how to use mixture density networks [24] to approximate the posterior for ABC calculations. In an ABC framework with a parametric exponential family, see [20,25] for the optimal choice of summary statistics. A local smoothing version of ABC is given in [4,26,27], ref. [28] take a basis function approach. Ref. [29] provides an estimation procedure when latent variables are present.

GBC provides an alternative to nonparametric Gaussian process-based surrogates which heavily rely on the informational contribution of each sample point and quickly become ineffective when faced with significant increases in dimensionality [30,31]. Furthermore, homogeneous GP models predict poorly [32]. Unfortunately, the consideration of each input location to handle these heteroskedastic cases results in analytically intractable predictive density and marginal likelihoods [33]. The smoothness assumption made by GP models hinders capturing rapid changes and discontinuities in the input–output relations. Popular attempts to overcome these issues include relying on the selection of kernel functions using prior knowledge about the target process [34]; splitting the input space into subregions so that, inside each of those smaller subregions, the target function is smooth enough and can be approximated with a GP model [35,36,37]; and learning spatial basis functions [38,39,40].

For low-dimensional θ, the simplest approach is to discretize the parameter space and the data space and provide a lookup table to approximate π( θ∣*y*). However, this approach is not scalable to high-dimensional θ. For practical cases, when the dimension of θ is high, we can use conditional independence structure present in the data to decompose the joint distribution into a product of lower-dimensional functions [41]. In machine learning, some approaches rely on such a decomposition [41,42,43]. Most of these approaches use KL divergence as a metric of closeness between the target distribution and the learned distribution. We propose to compute quantiles with the 1-Wasserstein distance as a metric of closeness between the target distribution and the learned distribution.

A natural approach to model the posterior distribution using a neural network is to assume that the parameters of the network are random variables and use MCMC techniques to model the posterior distribution over the parameters [44]. A slightly different approach is to assume that only the weights of the last output layer of a neural network are stochastic [45,46]. GBC provides a natural alternative to these methods.

The rest of the paper is outlined as follows. Section 1 provides a review of the existing literature. Section 2 describes our GBC algorithm. Section 3 describes quantile neural networks (QNNs). Section 4 describes the use of quantile neural networks for Bayesian computations. Section 5 provides two real-world applications from a traffic flow prediction problem and a satellite drag dataset. Finally, Section 6 concludes with directions for future research. There are a number of advantages to our approach. First, it is density-free as we are only estimating quantile functions. Secondly, it can calculate functionals of interest in an efficient manner; see [1].

## 2. Generative Bayesian Computation (GBC)

Bayesian inference requires samples from the posterior distribution of the parameters given the data. We use the following generic notation.

$$\begin{array}{cc} y= & \;\;\mathrm{outcome}\;\mathrm{of}\;\mathrm{interest}\\ \theta = & \;\;\mathrm{parameters}\\ \tau = & \;\;\mathrm{base}\;\mathrm{distribution}. \end{array}$$

To fix notation, let Y denote a locally compact metric space of signals, denoted by *y*, and B(Y) the Borel σ-algebra of Y. Let λ be a measure on the measurable space of signals (Y,B(Y)). Let P(dy|θ) denote the conditional distribution of signals given the parameters. When a likelihood p(y|θ) is available w.r.t. the measure λ, we write P(dy|θ)=p(y|θ)λ(dy).

Let Θ denote a locally compact metric space of admissible parameters (a.k.a. hidden states and latent variables z∈Z) and B(Θ) the Borel σ-algebra of Θ. Let μ be a measure on the measurable space of parameters (Θ,B(Θ)). Let Π(dθ|y) denote the conditional distribution of the parameters given the observed signal *y* (a.k.a., the posterior distribution). In many cases, Π is absolutely continuous with density π such that

Π(dθ|y)=π(θ|y)μ(dθ)

We will write Π(dθ)=π(θ)μ(dθ) for the prior density π when available.

Specifically, suppose that *Y* and θ are generated from a model M and prior to π. This generative approach will allow us to create a training dataset $\left(\theta^{\left(i\right)},y^{\left(i\right)}\right)$ for which we can construct our transport map from a base distribution τ.

Let $Y=y_{\mathrm{obs}}$ denote the observed output. The goal is to be able to draw samples from

$$\theta^{\left(i\right)}∼\pi \left(\theta |Y=y_{\mathrm{obs}}\right)\;\;\mathrm{for}\;\;1\leq i\leq N.$$

Our framework allows for likelihood and density free models. In the case of likelihood-free models, the output is simply specified by a map (a.k.a. forward equation).

**Noise Outsourcing Theorem**: In many practical applications, the observed *y*s are high-dimensional, and we can improve the performance of the deep neural network by using a summary statistic S(y). *G*ere $S:{ℜ}^{N}\to {ℜ}^{k}$ is a *k*-dimensional sufficient statistic, which is a lower-dimensional representation of *y*. The summary statistic is a sufficient statistic in the Bayes sense [3]. The main idea is to use a deep neural network to approximate the posterior mean $E_{\pi }\left(\theta |y\right)$ as the optimal estimate of S(y).

We propose a non-parametric generative approach to constructing a conditional distribution $P_{Y|X}$ for a given value of the predictor *X*, we estimate a function G(τ,x) where τ random variable from the reference distribution, e.g., a uniform, such that $G\left(\tau ,x\right)∼P_{Y|X=x}$. To Sample from $P_{Y|X}$, we simply generate τ from the reference distribution and evaluate G(τ,x). This provides a connection between the conditional distribution estimation and the generalized non-parametric regression. The underpinning is the noise outsourcing theorem ([47], [Theorem 5.10]) which states that any random variable can be represented as a function of another random variable and a noise term. We propose to use a quantile neural network to learn the function G(τ,x), rather than conditional GANs. Specifically, our goal is to find a function $G:R^{d}\times X\to Y$ such that the conditional distribution of G(τ,X), given X=x is the same as the conditional distribution of *Y* given X=x. Since τ is independent of *X*, it is equivalent of finding a *G* such that $G\left(\tau ,x\right)∼P_{Y|X=x}$, for any x∈X. The existence of *G* is guaranteed by the noise outsourcing theorem [47], namely,

$$\left(X,Y\right)=\left(X,G\left(\tau ,X\right)\right)\;a.s.,\;\mathrm{and}\;G\left(\tau ,x\right)∼P_{Y|X=x}\;\mathrm{for}\;\mathrm{any}\;x\in X.$$

This lemma also provides a unified view of conditional distribution estimation and non-parametric regression. To see this, it is useful to reverse the order and write

Y∣X=x∼G(τ,x)foranyx∈X.

This shows that the problem of finding *G* is equivalent to a non-linear non-parametric regression.

Now, we apply the noise outsourcing theorem to the problem of calculating a joint distribution Y,θ and conditional distribution θ∣Y, required for Bayesian inference.

**Theorem** **1.** *If (Y,θ) are random variables in a Borel space (Y,Θ) then there exists an r.v. τ∼U(0,1) which is independent of Y and a function G:[0,1]×Y→Θ such that*

$$\left(Y,\theta \right)\overset{a.s.}{=}\left(Y,G\left(Y,\tau \right)\right)$$

Hence the existence of *G* follows from the noise outsourcing theorem [47]. Moreover, if there is a statistic S(Y) with Y⊥⊥θ|S(Y), then

$$\left(\theta |Y=y\right)\overset{a.s.}{=}G\left(S\left(y\right),\tau \right).$$

The role of S(Y) is equivalent to the ABC literature. It performs dimension reduction in *n*, the dimensionality of the signal.

Assuming that we have fitted the deep neural network, *G*, to the training data, we can use the estimated inverse map to evaluate at new *y* and τ to obtain a set of posterior samples for any new *y* by interpolation and evaluating the map

(1) $$\theta \overset{D}{=}{\hat{G}}_{N}\left(S\left(y\right),\tau \right)\;,\;\;\mathrm{where}\;\;y=\left(y_{1},\ldots ,y_{n}\right)$$

where ${\hat{G}}_{N}$ denotes the estimated map. There are many well-known functional approximation rates for classes of Hölder-smooth functions and deep architectures for *G*. Ref. [48] provides conditional quantile results, for deep ReLU networks.

A caveat is how to choose *G* and how well the deep neural network interpolate for the observed input $y_{\mathrm{obs}}$. There is also flexibility in choosing the distribution of τ; for example, τ can also be a high-dimensional vector of Gaussians and essentially provide a mixture-Gaussian approximation for the set of posterior. GBC in a simple way is using pattern matching to provide a look-up table for the map from *y* to θ. Bayesian computation has then being replaced by the optimization performed by Stochastic Gradient Descent (SGD). Thus, we can sequentially update *G* using SGD step as new data arrives. MCMC, in comparison, is computationally expensive and needs to be re-run for any new data point. In our examples, we discuss choices of architectures for *G* and *S*. Specifically, we propose cosine-embedding for transforming τ.

**GBC Algorithm:** A necessary condition is the ability to simulate the parameters, latent variables, and data processes. This generates a (potentially large) triple

$${\left\{y^{\left(i\right)},\theta^{\left(i\right)},\tau^{\left(i\right)}\right\}}_{i=1}^{N},$$

By construction, the posterior distribution can be characterized by the von Neumann inverse CDF map. For θ∈ℜ we have

$$\theta \overset{D}{=}F_{\theta |y}^{-1}\left(\tau \right),\;\;\mathrm{where}\;\;\tau ∼U\left(0,1\right)$$

Given a new base draw τ, we then simply evaluate the following posterior map:

$$\theta \overset{D}{=}G\left(S\left(y\right),\tau \right)$$

When the base draw τ is uniform of same dimension as the parameter vector, the map *G* is precisely the inverse posterior CDF. This characterizes the posterior distribution p(θ|y) of interest. As simulation and fitting deep neural networks are computationally cheap, we typically choose *N* to be of order $10^{6}$ or more.

In many cases, we can estimate a summary statistic, *S* also via deep learning, and we write the inverse CDF as a composite map, and we have to fit

$$\theta^{\left(i\right)}=G\left(S\left(y^{\left(i\right)}\right),\tau^{\left(i\right)}\right)\;\;\mathrm{where}\;\;F_{\theta |y}^{-1}=G\circ S$$

There are many variations in the architecture design and construction. It is useful to interpret *S* as a summary statistic that provides a dimension reduction in the *y* space.

**Figure 1:**
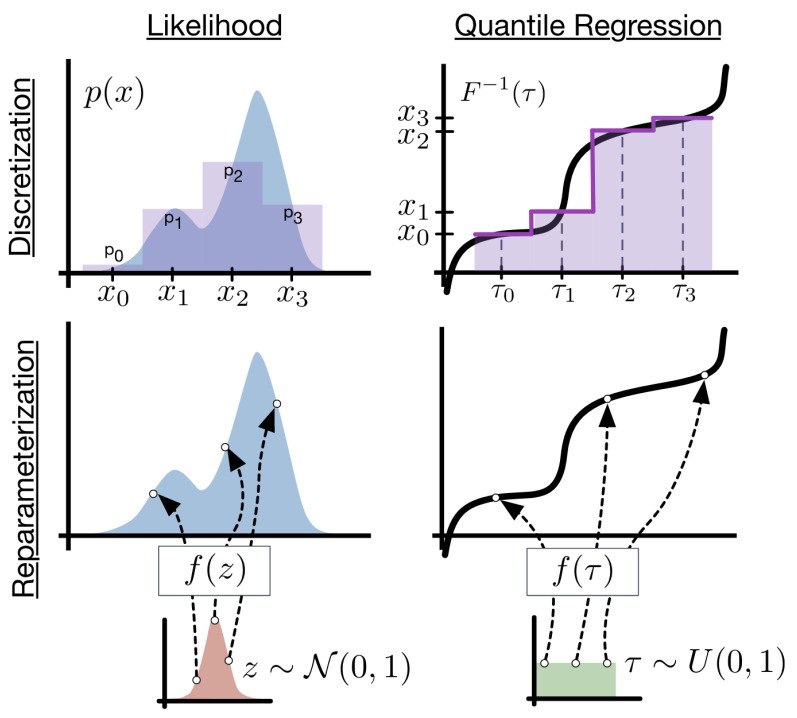
Deep Quantile Neural Network. Source: [6].

There is flexibility in the choice of base distribution. For example, we can replace τ with a different distribution from which we can easily sample. One example is a multi-variate normal, proposed for diffusion models [49]. The dimensionality of the normal can be large. The main insight is that you can solve a high-dimensional least squares problem with non-linearity using stochastic gradient descent. Deep quantile NNs provide a natural candidate for deep learners. Ref. [5] show that implicit quantile networks can be used for distributional reinforcement learning and has proposed the use of quantile regression as a method to minimize 1-Wasserstein in the univariate case when approximating using a mixture of Dirac functions. Figure 1 illustrates the difference in using a quantile objective versus pure density simulation based on the latent variable approach.

Hence, even if $y_{\mathrm{obs}}$ is not in the simulated training data set, we can still learn the posterior map of interest. The Kolmogorov–Arnold theorem says that any multivariate function can be expressed this way. So in principle if sample size is large enough we can learn the manifold structure in the parameters for any arbitrary non-linearity. As the dimension of the data *y* is large, in practice, this requires providing an efficient architecture. The following Algorithm 1 provides an algorithmic summary of Generative Bayesian Computation (GBC).
**Algorithm 1** Generative Bayesian Computation (GBC)1:Simulate $\theta^{\left(i\right)}∼\pi \left(\theta \right)$ and ${\left(y^{\left(i\right)}|\theta^{\left(i\right)}\right)}_{1\leq i\leq N}∼p\left(y|\theta \right)$ or $y^{\left(i\right)}=f\left(\theta^{\left(i\right)}\right)$ and $\tau^{\left(i\right)}∼p\left(\tau \right)$.2:Learn a summary statistic S(y) using a deep neural network.3:Train *G* using the simulated dataset for i=1,…N, via$$\theta^{\left(i\right)}=G\left(S\left(y^{\left(i\right)}\right),\tau^{\left(i\right)}\right)$$4:Given $y_{\mathrm{obs}}$, calculate a sample from π(θ∣y) with a new base draw τ∼p(τ) by$$\theta \overset{D}{=}{\hat{G}}_{N}\left({\hat{S}}_{N}\left(y_{\mathrm{obs}}\right),\tau \right)$$
using the trained architectures $\left({\hat{G}}_{N},{\hat{S}}_{N}\right)$.

In the case when *y* is high-dimensional, and we can learn a summary statistic S(y) using a deep neural network as an estimate of the posterior mean from the training data, namely using

$${\hat{S}}_{N}\left(y\right)=\hat{\theta }\left(y\right)=E_{\pi }\left(\theta |y\right).$$

### Summary Statistics S(y)

Given *y*, the posterior density is denoted by π(θ∣y). Here, $y=(y_{1},\ldots ,y_{n})$ is high-dimensional. Moreover, we need the set of posterior probabilities $\pi_{\theta |y}\left(\theta \in B|y\right)$ for all Borel sets *B*. Hence, we need dimension reduction for *y*. The whole idea is to estimate “maps” (a.k.a. transformations/feature extraction) of the output data *y*; thus, it is reduced to uniformity.

There is a nice connection between the posterior mean and the sufficient statistics, especially the minimal sufficient statistics in the exponential family. If there exists a sufficient statistic $S^{*}$ for θ, then [3] shows that for almost every *y*, $\pi \left(\theta |y\right)=\pi (\theta |S^{*}\left(y\right))$, and further $S\left(y\right)=E_{\pi }\left(\theta |y\right)=E_{\pi }\left(\theta |S^{*}\left(y\right)\right)$ is a function of $S^{*}\left(y\right)$. In the special case of an exponential family with minimal sufficient statistic $S^{*}$ and parameter θ, the posterior mean $S\left(y\right)=E_{\pi }\left(\theta |y\right)$ is a one-to-one function of $S^{*}\left(y\right)$, and thus is a minimal sufficient statistic. Deep learners are good interpolators (folklore theorem of machine learning).

Hence, the set of posteriors π(θ∣y) is characterized by the distributional identity $\theta \overset{D}{=}G\left(S\left(y\right),\tau \right),\;\;\mathrm{where}\;\;\tau ∼U\left(0,1\right)$.

We can construct *S* using deep learning. Ref. [22] shows that the optimal choice of $S\left(y\right)=E_{\pi }\left(\theta |y\right)$, namely the posterior mean namely $\hat{\theta }\left(y\right)$.

**Kolmogorov result on summary statistic:** Let S(y) be a sufficient summary statistic in the Bayes sense [3], if for every prior π

$$f_{B}\left(y\right):=\pi_{\theta |y}\left(\theta \in B|y\right)=\pi_{\theta |s(y)}\left(\theta \in B|s\left(y\right)\right).$$

Then we need to use our pattern matching dataset $\left(y^{\left(i\right)},\theta^{\left(i\right)}\right)$ which is simulated from the prior and forward model to “train” the set of functions $f_{B}\left(y\right)$, where we pick the sets B=(−∞,q] for a quantile *q*. Hence, we can then interpolate the mapping to the observed data.

The notion of a summary statistic is prevalent in the ABC literature and is closely related to the notion of a Bayesian sufficient statistic $S^{*}$ for θ, then [3]), for almost every *y*,

$$\pi \left(\theta |Y=y\right)=\pi (\theta |S^{*}\left(Y\right)=S^{*}\left(y\right))$$

Furthermore, $S\left(y\right)=E\left(\theta |Y=y\right)=E_{\pi }\left(\theta |S^{*}\left(Y\right)=S^{*}\left(y\right)\right)$ is a function of $S^{*}\left(y\right)$. In the case of exponential family, we have $S\left(Y\right)=E_{\pi }\left(\theta |Y\right)$ is a one-to-one function of $S^{*}\left(Y\right)$, and thus is a minimal sufficient statistic.

Sufficient statistics are generally kept for parametric exponential families, where S(·) is given by the specification of the probabilistic model. However, many forward models have an implicit likelihood and no such structures. The generalization of sufficiency is a summary statistics (a.k.a. feature extraction/selection in a neural network). Hence, we make the assumption that there exists a set of features such that the dimensionality of the problem is reduced.

Learning *S* can be achieved in a number of ways. First, *S* is of fixed dimension $S\in {ℜ}^{s}$ even though $y=(y_{1},\ldots y_{n})$. Typical architectures include Auto-encoders and traditional dimension reduction methods. Ref. [13] propose to use a theoretical result of Brillinger methods to perform a linear mapping S(y)=Wy and learn *W* using PLS. Ref. [50] extend this to IV regression and casual inference problems. A key result of [14] shows that we can use linear SGD methods and partial least squares to find $\hat{B}$.

To learn the feature selection variables S(y), ref. [22] propose to use a deep learner to approximate the posterior mean $E_{\pi }\left(\theta |y\right)$ as the optimal estimate of S(y). Specifically, they construct a minimum squared error estimator $\hat{\theta }\left(y\right)$ from a large simulated dataset and calculate

$$\min_{\psi }\frac{1}{N}\sum_{i=1}^{N}{∥S\left(y^{\left(i\right)}\right)-\theta^{\left(i\right)}∥}_{2}^{2}$$

where *S* denotes a DNN. The resulting estimator $\hat{\theta }\left(y\right)={\hat{S}}_{N}\left(y^{\left(i\right)}\right)$ approximates the feature vector S(y).

Together with the following architecture for the summary statistic neural network

$$\begin{array}{cc} S^{\left(1\right)}= & ReLU\left(W^{\left(0\right)}S^{\left(0\right)}+b^{\left(0\right)}\right)\\ S^{\left(2\right)}= & ReLU\left(W^{\left(1\right)}S^{\left(1\right)}+b^{\left(1\right)}\right)\\ \; & \vdots \\ S^{\left(L\right)}= & ReLU\left(W^{\left(L-1\right)}S^{\left(L-1\right)}+b^{\left(L-1\right)}\right)\\ \hat{\theta }= & W^{\left(L\right)}H^{\left(L\right)}+b^{\left(L\right)}, \end{array}$$

where $S^{\left(0\right)}=\theta$ is the input, and $\hat{\theta }$ is the summary statistic output.

**Figure 2:**
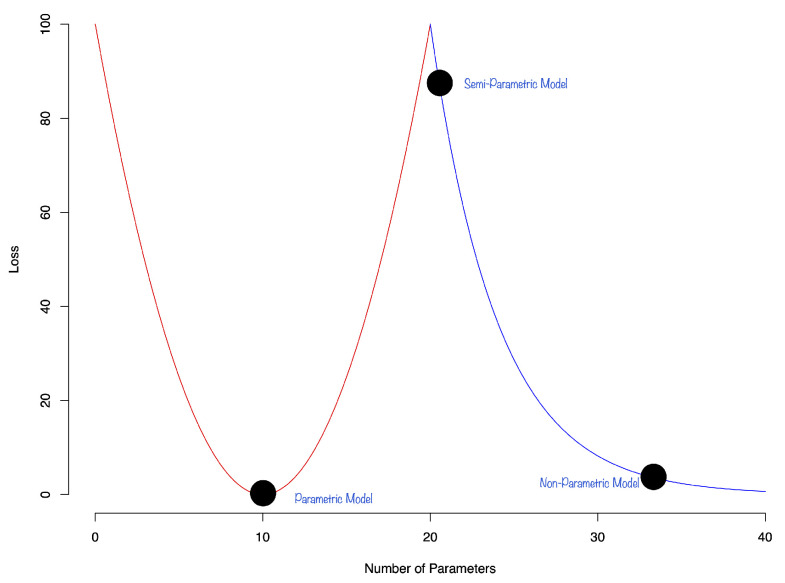
Stylized double descent curve.

**Double Descent:** There is still the question of approximation and the interpolation properties of a DNN. In general, deep learners provide good representations of multi-variate functions and are good interpolators. Recent research on interpolation properties of quantile neural networks were recently studied by [51,52,53]. See also [54,55]. They demonstrate that the generalization error of a DNN exhibits a double descent phenomenon. The authors showed that the test error of the estimator can decrease as the number of parameters increases. The phenomenon of double decent is shown in Figure 2:

The first part of the curve is the classical u-shaped bias-variance trade-off. The second part of the curve is the double descent phenomenon that shows that the test error of the estimator can decrease as the model becomes over-parametrized. This phenomenon was later observed in the context of deep learning [56]. The authors showed that the test error of the estimator can decrease as the number of parameters increases. Ref. [57] discuss theoretical bounds for generalization error for overparametrized models. They show that the generalization error of a model can be bounded by the number of parameters in the model.

The other two sources of error are the training error and the functional approximation error. The training error is a function of *N* and the functional approximation error depends on the smoothness properties of the underlying posterior and the flexibility of the neural networks used to approximate the posterior.

**ABC** Approximate Bayesian Computation (ABC) is a generative method for obtaining samples from the posterior distribution. It is a common approach in cases when likelihood is not available, but samples can be generated from some model. The ABC rely on comparing summary statistic calculated from data $s_{\mathrm{obs}}$ and of from observed output. We will denote a training sample by input-output pairs $\left(\theta^{\left(i\right)},Y^{\left(i\right)}\right)$. The ABC posterior requires a kernel, a dimensionality reducing summary statistic S(y) and a tolerance level ϵ with a tilted-posterior defined by

$$\pi_{ABC}^{\epsilon }\left(\theta |Y=y_{\mathrm{obs}}\right)=\frac{1}{m_{\epsilon }\left(y_{\mathrm{obs}}\right)}\int K_{\epsilon }\left(S\left(y\right)-y_{\mathrm{obs}}\right)\lambda_{\theta }\left(dy\right)\pi \left(d\theta \right).$$

Here $y=(y_{1},\ldots ,y_{n})$ is high dimensional. Hence, the need for a *k*-dimensional summary statistic $S:{ℜ}^{n}\to {ℜ}^{k}$ where *k* is fixed.

This ensures that the mean of the ABC posterior matches that of the posterior of interest. Furthermore, under a uniform kernel $K=I(|S\left(y\right)-s_{\mathrm{obs}}|<\epsilon )$ we show convergence

$$\pi_{ABC}^{\epsilon }\left(\theta |Y=y_{\mathrm{obs}}\right)\to \pi \left(\theta |Y=y_{\mathrm{obs}}\right).$$

Specifically, the ABC posterior is given by

$$\begin{array}{cc} \pi_{ABC}^{\epsilon }\left(\theta |y\right) & =\frac{1}{m_{\epsilon }\left(y_{\mathrm{obs}}\right)}\int_{\Theta }I(|S\left(y\right)-s_{\mathrm{obs}}|<\epsilon )\delta \left(y-f\left(\theta \right)\right)\pi \left(\theta \right)dyd\theta =\pi (\theta ||S\left(y\right)-s_{\mathrm{obs}}\left|<\epsilon \right)\\ \; & \to \pi \left(\theta |Y=y_{\mathrm{obs}}\right),\;\mathrm{where}\;S\left(y\right)=\hat{\theta }\left(y\right)=E_{\pi }\left(\theta |y\right). \end{array}$$

A deep NN can effectively learn a good approximation to the posterior mean $E_{\pi }$(θ∣*y*) by using a large data set ${\left(y^{\left(i\right)},\theta^{\left(i\right)}\right)}_{i=1}^{N}∼\pi \times M$ and solving the $\ell_{2}$ — minimization problem

$$\arg\min_{\psi }\frac{1}{N}\sum_{i=1}^{N}{∥S\left(y^{\left(i\right)}\right)-\theta^{\left(i\right)}∥}^{2}.$$

The resulting estimator $\hat{\theta }\left(y\right)=\hat{S}\left(y\right)$ approaches S(y). This still leaves the problem of simulating a training data set to learn the ABC posterior, which requires the rejection sampling. Our approach, on the other hand, rectifies this issue. Our approach directly evaluates the network on $y_{\mathrm{obs}}$ and relies on good generalization error, a.k.a. interpolation.

Conventional ABC methods suffers from the main drawback that the samples do not come from the true posterior, but an approximate one, based on the ϵ-ball approximation of the likelihood, which is a non-parametric local smoother. Theoretically, as ϵ goes to zero, you can guarantee samples from the true posterior. However, the number of sample required is prohibitive. Our method circumvents this by replacing the ball with a deep learning generator and directly model the relationship between the posterior and a baseline uniform Gaussian distribution. Our method is also not a density approximation, as many authors have proposed. Rather, we directly use $L_{2}$ methods and Stochastic Gradient Descent to find transport map from θ to a uniform or a Gaussian random variable. The equivalent to the mixture of Gaussian approximation is to assume that our baseline distribution is high-dimensional Gaussian. Such models are called the diffusion models in literature. Full Bayesian computations can then be reduced to high-dimensional $L_{2}$ optimization problems with a carefully chosen neural network.

## 3. Bayes Quantile Neural Networks

One can show that quantile models are direct alternatives to other Bayes computations. Specifically, given F(y), a non-decreasing and continuous from right function. We define

$$Q_{\theta |y}\left(u\right):=F_{\theta |y}^{-1}\left(u\right)=\inf\left(y:F_{\theta |y}\left(y\right)\geq u\right),$$

which is nondecreasing and continuous from the left.

Now, let g(y) to be a non-decreasing and continuous from left with

$$g^{-1}\left(z\right)=\sup\left(y:g(y)\leq z\right)$$

Then, the transformed quantile has a compositional nature, namely

$$Q_{g(Y)}\left(u\right)=g\left(Q\left(u\right)\right)$$

Hence, quantiles act as superposition (a.k.a. deep Learner).

This is best illustrated in the Bayes learning model. We have the following result updating prior to posterior quantiles known as the conditional quantile representation

$$Q_{\theta |Y=y}\left(u\right)=Q_{Y}\left(v\right)\;\;\mathrm{where}\;\;v=Q_{F(\theta )|Y=y}\left(u\right)$$

To compute *v* we use

$$u=F_{F(\theta )|Y=y}\left(v\right)=P\left(F\left(\theta \right)\leq v|Y=y\right)=P\left(\theta \leq Q_{\theta }\left(v\right)|Y=y\right)=F_{\theta |Y=y}\left(Q_{\theta }\left(v\right)\right)$$

Ref. [58] also shows the following probabilistic property of quantiles

$$\left(\theta |Y=y\right)=Q_{\theta |Y=y}\left(F_{\theta |y}\left(\theta \right)\right).$$

Hence, we can increase the efficiency by ordering the samples of θ and the baseline distribution, since the mapping being the inverse CDF is monotonic.

### 3.1. Learning Quantiles

The 1-Wasserstein distance is the $\ell_{1}$ metric of the inverse distribution function. It is also known as earth mover’s distance and can be calculated using order statistics [59]. For quantile functions $F_{U}^{-1}$ and $F_{V}^{-1}$ the 1-Wasserstein distance is given by

$$W_{1}\left(F_{U}^{-1},F_{V}^{-1}\right)=\int_{0}^{1}\left|F_{U}^{-1}\left(\tau \right)-F_{V}^{-1}\left(\tau \right)\right|d\tau .$$

Ref. [60] shows that Wasserstein GANs outperform vanilla GAN due to the improved quantile metric $q=F_{U}^{-1}\left(\tau \right)$ minimize the expected quantile loss

$$E_{U}\left[\rho_{\tau }\left(u-q\right)\right]$$

Quantile regression can be shown to minimize the 1-Wasserstein metric. A related loss is the quantile divergence,

$$q\left(U,V\right)=\int_{0}^{1}\int_{F_{U}^{-1}\left(q\right)}^{F_{V}^{-1}\left(q\right)}\left(F_{U}\left(\tau \right)-q\right)dqd\tau .$$

The quantile regression likelihood function is an asymmetric function that penalizes overestimation errors with weight τ and underestimation errors with weight 1−τ. For a given input-output pair (x,y), and the quantile function f(x,θ), parametrized by θ, the quantile loss is $\rho_{\tau }\left(u\right)=u\left(\tau -I\left(u<0\right)\right)$, where u=y−f(x). From an implementation point of view, a more convenient form of this function is

$$\rho_{\tau }\left(u\right)=\max\left(u\tau ,u\left(\tau -1\right)\right).$$

Given training data ${\left\{x_{i},y_{i}\right\}}_{i=1}^{N}$, and a quantile τ, the loss is

$$L_{\tau }\left(\theta \right)=\sum_{i=1}^{N}\rho_{\tau }\left(y_{i}-f\left(\tau ,x_{i},\theta \right)\right).$$

Further, we empirically found that adding a means-squared loss to this objective function improves the predictive power of the model, thus the loss function, we use is

$$\alpha L_{\tau }\left(\theta \right)+\frac{1}{N}\sum_{i=1}^{N}{\left(y_{i}-f\left(x_{i},\theta \right)\right)}^{2}.$$

One approach to learn the quantile function is to use a set of quantiles $0<\tau_{1}<\tau_{2},\ldots ,\tau_{K}<1$ and then learn *K* quantile functions simultaneously by minimizing

$$L\left(\theta ,\tau_{1},\ldots ,\tau_{K}\right)=\frac{1}{NK}\sum_{i=1}^{N}\sum_{k=1}^{K}\rho_{\tau_{k}}\left(y_{i}-f_{\tau_{k}}\left(x_{i},\theta_{k}\right)\right).$$

The corresponding optimization problem of minimizing L(θ) can be augmented by adding a non-crossing constraint.

$$f_{\tau_{i}}\left(x,\theta_{i}\right)<f_{\tau_{j}}\left(x,\theta_{j}\right),\;\forall X,\;i<j.$$

The non-crossing constraint has been considered by several authors, including [61,62].

**Cosine Embedding for τ:** We will use the following architecture design to learn an inverse CDF (quantile function), namely $F^{-1}\left(\tau ,y\right)=f_{\theta }\left(\tau ,y\right)$, a kernel embedding which augments the input space to the network. The quantile function is represented as a superposition of two other functions $F^{-1}\left(\tau ,y\right)=f_{\theta }\left(\tau ,y\right)=g\left(\psi \left(y\right)\circ \phi \left(\tau \right)\right)$ where ∘ is the element-wise multiplication operator. Both functions *g* and ψ are feed-forward neural networks and ϕ is a cosine embedding transform. To avoid overfitting, we use a sufficiently large training dataset; see [5] in a reinforcement learning context. Dimension reduction (a.k.a. feature extraction) will draw on methods used in ABC; see [4] for an approach using semiautomatic approximate to the sufficient statistic.

To learn an inverse CDF (quantile function) $F^{-1}\left(\tau ,y\right)=f_{\theta }\left(\tau ,y\right)$ we will use a kernel embedding trick and augment the predictor space. The quantile function is a superposition for two other functions

$$F^{-1}\left(\tau ,y\right)=f_{\theta }\left(\tau ,y\right)=g\left(\psi \left(y\right)\circ \phi \left(\tau \right)\right),$$

where ∘ is the element-wise multiplication operator. Both functions *g* and ψ are feed-forward neural networks. ϕ is a cosine embedding. To avoid over-fitting, we use a sufficiently large training dataset; see [5] in a reinforcement learning context.

Let *g* and ψ be feed-forward neural networks and ϕ a cosine embedding given by

$$\phi_{j}\left(\tau \right)=\mathrm{ReLU}\left(\sum_{i=0}^{n-1}\cos\left(\pi i\tau \right)w_{ij}+b_{j}\right).$$

We now illustrate our approach with a simple synthetic dataset.

### 3.2. Synthetic Data

Consider a synthetic data generated from the model

$$\begin{array}{cc} x & ∼U(-1,1)\\ y & ∼N(\sin(\pi x)/(\pi x),\exp(1-x)/10). \end{array}$$

The true quantile function is given by

$$f_{\tau }\left(x\right)=\sin\left(\pi x\right)/\left(\pi x\right)+\Phi^{-1}\left(\tau \right)\sqrt{\exp(1-x)/10},$$

**Figure 3:**
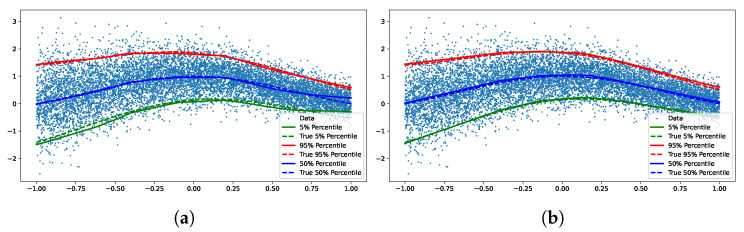
We trained both implicit and explicit networks on the synthetic data set. The explicit network was trained for three fixed quantiles (0.05, 0.5, 0.95). We see no empirical difference between the two. (**a**) Implicit quantile network. (**b**) Explicit quantile network.

We train two quantile networks, one implicit and one explicit. The explicit network is trained for three fixed quantiles (0.05, 0.5, 0.95). Figure 3 shows fits by both of the networks, we see no empirical difference between the two.

## 4. Bayes with Quantiles

### Normal–Normal Bayes Learning: Wang Distortion

For the purpose of illustration, we consider the normal–normal learning model. We will develop the necessary quantile theory to show how to calculate posteriors and expected utility without resorting to densities. Also, we show a relationship with Wang’s risk distortion measure as the deep learning that needs to be learned.

Specifically, we observe the data $y=(y_{1},\ldots ,y_{n})$ from the following model:

$$\begin{array}{cc} y|\theta & ∼N(\theta ,\sigma^{2})\\ \theta & ∼N(\mu ,\alpha^{2}) \end{array}$$

Hence, the summary (sufficient) statistic $S\left(y\right)=\bar{y}$. A remarkable result due to Brillinger, shows that we can learn *S* independent of *G* simply via OLS.

Given observed samples $y=(y_{1},\ldots ,y_{n})$, the posterior is then θ∣*y*∼*N*$\left(\mu_{*},\sigma_{*}^{2}\right)$ with

$$\mu_{*}=\left(\sigma^{2}\mu +\alpha^{2}s\right)/t,\;\sigma_{*}^{2}=\alpha^{2}\sigma^{2}/t,$$

where

$$t=\sigma^{2}+n\alpha^{2}\;\;\mathrm{and}\;\;s\left(y\right)=\sum_{i=1}^{n}y_{i}$$

The posterior and prior CDFs are then related via the Wang distortion function

$$1-\Phi \left(\theta ,\mu_{*},\sigma_{*}\right)=g\left(1-\Phi \left(\theta ,\mu ,\alpha^{2}\right)\right),$$

where Φ is the normal distribution function. Here,

$$g\left(p\right)=\Phi \left(\lambda_{1}\Phi^{-1}\left(p\right)+\lambda \right),$$

where

$$\lambda_{1}=\frac{\alpha }{\sigma_{*}}\;\;\mathrm{and}\;\;\lambda =\alpha \lambda_{1}\left(s-n\mu \right)/t.$$

The proof is relatively simple and is as follows:

$$\begin{array}{cc} g(1-\Phi \left(\theta ,\mu ,\alpha^{2}\right)) & =g\left(\Phi \left(-\theta ,\mu ,\alpha^{2}\right)\right)=g\left(\Phi \left(-\frac{\theta -\mu }{\alpha }\right)\right)\\ \; & =\Phi \left(\lambda_{1}\left(-\frac{\theta -\mu }{\alpha }\right)+\lambda \right)=1-\Phi \left(\frac{\theta -(\mu +\alpha \lambda /\lambda_{1})}{\alpha /\lambda_{1}}\right) \end{array}$$

with hyperparameters

$$\sigma_{*}=\alpha /\lambda_{1},\;\lambda_{1}=\frac{\alpha }{\sigma_{*}}$$

and

$$\mu_{*}=\mu +\alpha \lambda /\lambda_{1},\;\lambda =\frac{\lambda_{1}\left(\mu_{*}-\mu \right)}{\alpha }=\alpha \lambda_{1}\left(s-n\mu \right)/t.$$

We now provide a numerical example.

Consider the normal–normal model with prior θ∼N(0,5) and likelihood y∼N(3,10). We generate n=100 samples from the likelihood and calculate the posterior distribution.

The posterior distribution calculated from the sample is then θ∣*y*∼*N*(3.28, 0.98).

**Figure 4:**
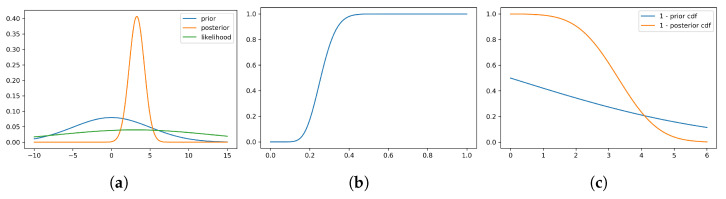
Density for prior, likelihood and posterior, distortion function and 1 - Φ for the prior and posterior of the normal–normal model. (**a**) Model for simulated data. (**b**) Distortion function *g*. (**c**) 1−Φ.

Figure 4 shows the Wang distortion function for the normal–normal model. The left panel shows the model for the simulated data, while the middle panel shows the distortion function, the right panel shows the 1−Φ for the prior and posterior of the normal–normal model.

## 5. Applications

### 5.1. Traffic Data

**Figure 5:**
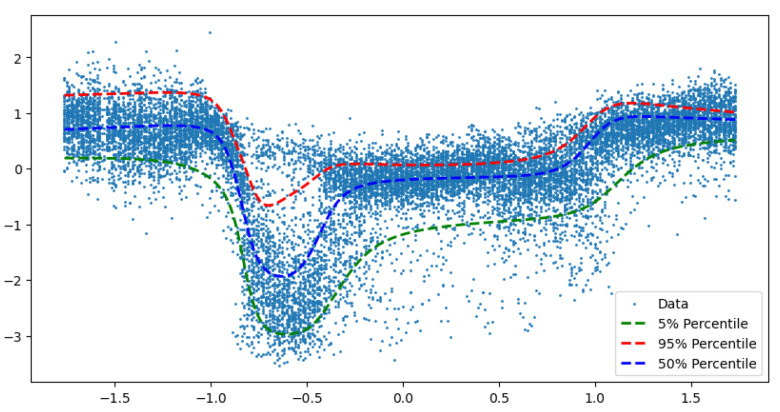
Implicit neural network for traffic speed observed on I-55 north-bound towards Chicago.

We further illustrate our methodology, using data from a sensor on interstate highway I-55. The sensor is eight miles from downtown Chicago on the northbound I-55 (near Cicero Ave), which is part of a route used by many morning commuters to travel from the suburbs of southwest to the city. As shown in Figure 5, the sensor is located 840 m downstream of an off-ramp and 970 m upstream from an on-ramp.

In a typical day traffic flow pattern on Chicago’s I-55 highway, where sudden breakdowns are followed by a recovery to free flow regime, we can see a breakdown in traffic flow speed during the morning peak period followed by speed recovery. The free flow regimes are usually of little interest to traffic managers. We also see that variance is low during the free-flow regime and high during the breakdown and recovery regimes.

We use the following architecture to model the implicit quantile function.

$$\begin{array}{cc} \tau_{1}= & \mathrm{ReLU}\left(w_{0}^{\left(1\right)}+\sum_{i=1}^{64}w_{i}^{\left(1\right)}\cos\left(i\pi \tau \right)\right)\\ x_{1}= & \mathrm{ReLU}\left(w_{0}^{\left(2\right)}+\sum_{i=1}^{64}w_{i}^{\left(2\right)}x_{i}\right)\\ z= & \mathrm{ReLU}\left(w_{0}^{\left(3\right)}+\sum_{i=1}^{64}w_{i}^{\left(3\right)}\tau_{i}x_{i}\right)\\ y_{1}= & \mathrm{ReLU}\left(w_{0}^{\left(4\right)}+\sum_{i=1}^{32}w_{i}^{\left(4\right)}x_{i}\right)\\ \hat{y}= & w_{0}^{\left(5\right)}+w_{i}^{\left(5\right)}x_{i}. \end{array}$$

Our earlier paper [63] analyzed the same data set using sequential Monte Carlo methods and can be used to compare the results.

We now consider a satellite drag example.

### 5.2. Satellite Drag

Accurate estimation of satellite drag coefficients in low Earth orbit (LEO) is vital for various purposes such as precise positioning (e.g., to plan manoeuvring and determine visibility) and collision avoidance.

Recently, 38 of 49 Starlink satellites launched by SpaceX on 3 February 2022 experienced an early atmospheric reentry caused by unexpectedly elevated atmospheric drag, an estimated $100 MM loss in assets. The launch of the SpaceX Starlink satellites coincided with a geomagnetic storm, which heightened the density of Earth’s ionosphere. This, in turn, led to an elevated drag coefficient for the satellites, ultimately causing the majority of the cluster to re-enter the atmosphere and burn up. This recent accident shows the importance of accurate estimation of drag coefficients in commercial and scientific applications [64].

The accurate determination of the drag coefficients is crucial for assessing and maintaining orbital dynamics by taking into account the drag force. Atmospheric drag is the primary source of uncertainty for objects in LEO. This uncertainty arises partially due to inadequate modeling of the interaction between the satellite and the atmosphere. Drag is influenced by various factors, including geometry, orientation, ambient and surface temperatures, and atmospheric chemical composition, all of which are dependent on the position of the satellite (latitude, longitude, and altitude).

Los Alamos National Laboratory developed the Test Particle Monte Carlo simulator to predict the movement of satellites in low earth orbit [65]. The simulator takes two inputs, the geometry of the satellite, given by the mesh approximation and seven parameters, which we list in Table 1 below. The simulator takes about a minute to run one scenario, and we use a dataset of one million scenarios for the Hubble Space Telescope [21]. The simulator outputs estimates of the drag coefficient based on these inputs, while considering uncertainties associated with atmospheric and gas–surface interaction models (GSI).

We use the data set of 1 million simulation runs provided by [2]. The data set has 1 million observations, and we use 20% for training and 80% for testing out-of-sample performance. The model architecture is given below. We use the Adam optimizer and a batch size of 2048, and train the model for 200 epochs.

Ref. [66] provides a survey of modern Gaussian process-based models for prediction and uncertainty quantification tasks. They compare five different models and apply them to the same Hubble data set we use in this section. We use two metrics to assess the quality of the model, namely RMSE, which captures predictive accuracy, and the continuous rank probability score (CRPS; [67,68]). Essentially, CRPS is the absolute difference between the predicted and observed cumulative distribution function (CDF). We used the degenerative distribution with the entire mass on the observed value (Dirac delta) to obtain the observed CDF. The lower the CRPS, the better.

Their best performing model is treed-GP has the RMSE of 0.08 and CRPS of 0.04, the worst performing model is the deep GP with approximate “doubly stochastic” variational inference that has the RMSE of 0.23 and the CRPS of 0.16. The best performing model in our experiments is the quantile neural network with RMSE of 0.098 and CRPS of 0.05, which is comparable to the top performer from the survey.

**Figure 6:**
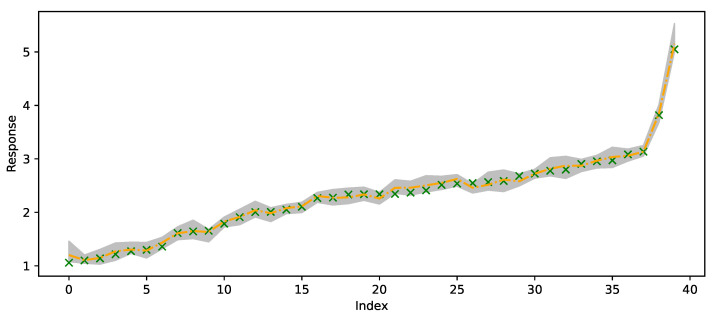
Randomly selected 40 out-of-sample observations from the satellite drag dataset. Green crosses are observed values, orange line is predicted 50% quantile and the gray region is the 95% credible prediction interval.

Figure 6 plots of the out-of-sample predictions for forty randomly selected responses (green crosses) and compares those to 50th quantile predictions (orange line) and 95% credible prediction intervals (gray region).

**Figure 7:**
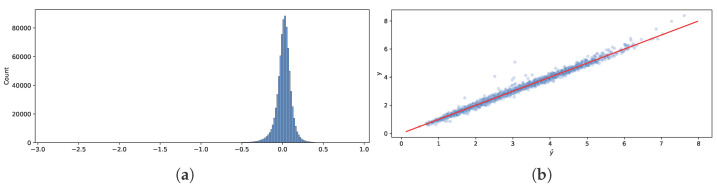
Comparison of out-of-sample predictions (50% quantiles) $\hat{y}$ and observed drag coefficients *y*. (**a**) Histogram of errors $\hat{y}-y$. (**b**) *y* vs. $\hat{y}$.

Figure 7a compares the out-of-sample predictions (50% quantiles) $\hat{y}$ and observed drag coefficients *y*. We can see that the histogram resembles a normal distribution centered at zero, with some “heaviness” on the left tail, meaning that for some observations, our model underestimates. The scattershot in Figure 7b shows that the model is more accurate for smaller values of *y* and less accurate for larger values of *y* and values of *y* around three.

**Figure 8:**
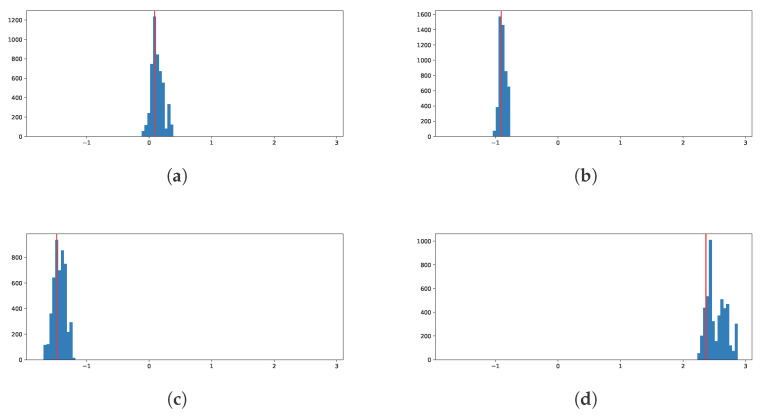
Posterior histograms for four randomly chosen out-of-sample response values. The vertical red line is the observed value of the response. (**a**) Observation 948127. (**b**) Observation 722309. (**c**) Observation 608936. (**d**) Observation 988391.

Finally, we show histograms of the posterior predictive distribution for four randomly chosen out-of-sample response values in Figure 8. We can see that the model concentrates the distribution around the true values of the response.

Overall, our model provides competitive performance to the state-of-the-art techniques used for predicting and uncertainty quantification (UQ) analysis of complex models, such as satellite drag. The model is able to capture the distribution of the response and provide accurate predictions. The model is also capable of providing uncertainty quantification in the form of credible prediction intervals.

## 6. Discussion

Generative Bayes computations is a simulation-based methodology that takes joint samples of observables and parameters as input and then applies nonparametric regression in a form of deep neural network by regressing θ on a nonlinear function *h* which is a function of dimensionality-reduced sufficient statistics of θ and a randomly generated stochastically uniform error. In its simplest form, *h*, can be identified with its inverse CDF.

One solution to the multi-variate case is to use autoregressive quantile neural networks. There are also many alternatives to the architecture design that we propose here. For example, auto-encoder [8,69] or implicit models; see [18,46,70]. There is also a link with indirect inference methods developed in [29,71,72,73]

There are many challenging future problems. The method can easily handle high-dimensional latent variables. However, designing the architecture for fixed high-dimensional parameters can be challenging, and we will leave this to future research. Having learned the non-linear map, when faced with the observed data $y_{\mathrm{obs}}$, one simply evaluates the non-linear map at newly generated uniform random values. Generative computations circumvent the need for methods like MCMC that require the density evaluations.

We also think that over-parameterization of the problem might lead to a useful model, although it might also lead to nonidentifiability of the weights in the regression. Two interesting approaches include the case where K>k and mixture models with Gaussian mixtures for τ.

## Figures and Tables

**Table 1:** Input parameters for the satellite drag simulator.

Parameter	Range
velocity [m/s]	[5500, 9500]
surface temperature [K]	[100, 500]
atmospheric temperature [K]	[200, 2000]
yaw [radians]	[−π,π]
pitch [radians]	[−π/2,π/2]
normal energy AC [unitless]	[0, 1]
tangential momentum AC [unitless]	[0, 1]

## Data Availability

Data are contained within the article.
